# Estimating the proportion of clinically diagnosed infectious and non-infectious animal diseases in Ganta Afeshum woreda, Eastern Tigray zone, Ethiopia

**DOI:** 10.1186/s13104-018-3158-3

**Published:** 2018-01-15

**Authors:** Mebrahtu Tedla, Mebrahtu Gebreselassie

**Affiliations:** Department of Biomedical Sciences, College of Veterinary Medicine and Animal Sciences, The University of Gondar, P.O. Box: 196, Gondar, Ethiopia

**Keywords:** Proportion, Livestock disease, Case diagnosis, Ganta-Afeshum

## Abstract

**Objective:**

This study was performed with the objective of identifying the proportion of emerging and endemic livestock diseases using cross sectional survey.

**Result:**

A total of 285 clinically diseased animals were presented to a veterinary clinic and diagnosed tentatively based on history, clinical sign, and simple laboratory diagnostics and from the study, actinomycosis (15.83%), mastitis (15%), tick infestation (10%), respiratory diseases (9.16%) and gastro intestinal parasitism (9.16%) were confirmed with higher proportion in large animals. Pasteurollosis (38, 31%), contagious ecthyma (12, 10%), tick infestation (9, 0%), mite infestation (9, 10%), sheep and goat pox (9, 10%), and gastrointestinal parasitism (9, 17%) were frequently encountered diseases in sheep and goat respectively. In equids, back sore, epizootic lymphangitis and lameness accounted a proportion of 22.95, 21.31, and 13.11% respectively. In conclusion, result of the present study showed that the proportion of livestock disease is high, and it affects the socioeconomic status of the local community in the study area as a result of mortality and production loss.

## Introduction

Livestock production is a principal means of achieving improved living standards in many regions of the developing world [1]. Livestock provide 20–30% of the gross domestic product and as much as 70% of cash income at the farmer level [2]. Livestock fulfills an important function in accumulating wealth, and serving as a store of value in the absence of formal financial institutions and other missing markets. In the case of smallholder mixed farming systems, livestock provides nutritious food, additional emergency and cash income, transportation, farm outputs and inputs, and fuels for cooking food [3]. Ethiopia constitutes an agrarian society with 85% of the populations livelihood primarily depend on traditional farming and animal husbandry practices [4]. At the household level, livestock plays a critical economic and social role in the lives of pastoralists, agro-pastoralists, and smallholder farm households. Ethiopia has the largest livestock inventories in Africa but the national and per capital production of livestock and livestock product, export earnings and per capital consumption of food from livestock origins have been low [5]. Various animal diseases have a paramount negative impact on productivity and fertility. For example, losses due to mortality and morbidity, loss of weight, low growth rate, poor fertility performance and decrease physical power [6]. The endemicity of livestock disease is increasing as a result of changing agro- ecological conditions and this enhances animal mobility from place to place. As a result, different pathogenic organisms get advantage from the dynamic state created by animal migration, and this increases pressure on scarce resources from all human and animal populations with more frequent contact for disease transmission [7]. The most important constraints to cattle productions are widespread diseases including viral, bacterial, and parasitic infestation, poor veterinary service, and lack of attention from government authorities [8]. Despite substantial international demand for Ethiopian meat and livestock products, exports are often hindered by endemic diseases which limit livestock productivity and agricultural development. Investigating the distribution and incidences of infectious and non-infectious diseases in a population assists in determining their importance and implementation of control campaigns [9]. Moreover, documenting the type and extent of major livestock health problems in a region is crucial for livestock owners, veterinarians and researchers and policy makers for development of heard health management strategies and selection of strategic interventions [10]. In the current study area, different infectious and non-infectious livestock diseases such as foot and mouth disease, epizootic lymphangitis, pasteurollosis, internal and external parasitism, mastitis, back sore, and abortion have been reported by veterinarians in the clinic. However, there is a paucity of information on the frequency of occurrence in the area and therefore, the objective of this study was to study the proportion of major livestock diseases in the Ganta Afeshum woreda.

## Main text

### Methods

#### Study area

This study was conducted from October 2013 to April 2014 in animals coming to veterinary clinic found at Adigrat town. Adigrat is located in the eastern zone of Tigray regional state which is situated at 115 kms North East of Mekelle, regional capital and 898 kms North of Addis Ababa. It is also located at a latitude of 14° 16 North and 39° 27 East and with an altitude of 1900–3000 m above sea level. The mean minimum and maximum annual rain fall is 400 and 600 mm respectively and the average temperature per year is 15 to 24 °C. Animal owners mainly farmers originated from different kebeles such as Buket, Genahti, Dibla, Maymesanu, Mitsnah wegebet, and from the town have presented their animal to veterinary clinic with a history of illness such as lack of appetite, decreased body condition, coughing, diarrhea, wound, lameness, bloat, nasal discharges etc.

#### Clinical diagnosis

The history of each animal was recorded before diagnosis and two main methodological approaches were used by the certified veterinarians to confirm the disease. Clinical cases with pathognomonic signs were considered as positive for the disease based on the clinical observation, physical diagnosis methods and history of the animal given by the owners, and considering the epidemiology of the diseases in the animal origin. Secondly, suspected clinical cases were confirmed using floatation and sedimentation techniques for identification of internal parasite eggs and simple gram staining technique for the identification of bacterial pathogens from blood and tissue samples. After confirmation, clinical cases were administered with recommended therapeutics for treatment. In addition, animal owners were asked using unstructured questionnaire survey to get information on the mortalities of cases and to identify the local names of mostly encountered livestock diseases and to incorporate into the body of knowledge.

#### Sampling strategies

during the study, a total of 285 animals were examined, particularly, 120 cattle, 61 equids, 75 sheep and 29 goat and each case was recorded with factors such as age, sex, breed, management system and body condition scores. Furthermore, a total of 100 livestock owners coming from the aforementioned kebele were asked using unstructured questionnaire survey which was translated into local language (Tigrigna).

#### Data management and analysis

Descriptive type of statistics was employed in order to show the incidence of all livestock diseases and in each species and factors associated with it, the incidence was calculated by dividing the number of animals’ positive for particular diseases by the total number of animals examined.

### Discussion and conclusion

#### Large animal diseases

A total of 120 cattle were examined and among the diagnosed diseases, actinomycosis (16%), mastitis (15%), tick infestation (10%), respiratory diseases (9%) and gastrointestinal parasitic diseases (9%) were the most commonly identified diseases. The proportion of bacilliary haemoglobin urea was 0.83%. Whereas, dystocia, paraphimosis, wound, lameness, and hoof over growth were 2% each and they were less frequent livestock diseases identified (Table 1).

**Table 1:** Incidence of major large animal diseases

Disease	Age		Sex		Breed		BCS			Management system			Overall proportion (%)
	Young (%)	Adult (%)	Male (%)	Female (%)	Local (%)	Cross (%)	Poor (%)	Medium (%)	Good (%)	Intensive (%)	Semi-intesive (%)	Extensiv e (%)	
Actinomycosis	36	63	68	31	57	42	57	47	5	15	21	63	16
Mastitis	–	100	–	100	44	55	22	44	33	50	28	22	15
Tick infestation	58	41	58	41	75	25	25	75	–	8	25	66	10
Respiratory disease	63	36	45	54	36	63	36	54	9.1	45	36	18	9
GIT helminthiasis	72	27	36	63	72	27	81	18	–	–	36	63	9
Mite infestation	44	55	33	66	77	22	77	22	–	–	33	66	7
Bloat	28	71	71	28	85	14	71	28	–	–	57	43	6
Black leg	100	–	71	28	42	57	–	14	85	–	71.4	28	6
RFM	–	100	–	100	80	20	–	80	20	20	80	–	4
Babesiosis	25	75	75	25	100	–	75	25	–	–	25	75	3
Actinobacillosis	–	100	66	33	100	–	33	66	–	–	33	66	3
Fashiolosis	66	33	66	33	100	–	100	–	–	–	–	100	3
Hoof over growth	–	100	100	–	–	100	–	50	50	50	50	–	2
Lameness	50	50	–	100	50	50	–	50	50	–	100	–	2
Wound	100	–	50	50	100	–	–	100	–	–	–	100	2
Paraphimosis	–	100	100	–	100	–	–	100	–	–	50	50	2
Dystocia	–	100	–	100	–	100	–	100	–	–	100	–	2
Baciliary hemoglobinuria	–	100	100	–	100	–	100	–	–	–	–	100	1

#### Sheep diseases

A total of 75 ovine cases were tentatively diagnosed in the clinic and pasteurollosis (38%), with high proportion in young animals (62%), contagious ecthyma (12%) and also with higher proportion in young animals (63.63%), and tick infestation (9%) were among the most frequently occurred ovine diseases in the study area. Cases such as pregnancy toxemia and wound were less commonly occurred cases with 1% proportion (Table 2).

**Table 2:** Incidence of small ruminant diseases

Species	Disease	Age		Sex		BCS			Management system			Overall proportion (%)
		Young (%)	Adult (%)	Male (%)	Female (%)	Poor (%)	Medium (%)	Good (%)	Intensive (%)	Semi-intensive (%)	Extensive (%)	
Ovine	Pasteurollo sis	62	38	34	65	31	55	14	–	14	86	38
	Orf	63	36	45	54	54	45	–	–	18	82	12
	Tick infestation	42	57	71	29	43	57	–	–	–	100	9
	Mite infestation	33	66	50	50	66	33	–	–	16	83	8
	Sheep and goat pox	66	33	33	66	33	66	–	–	16	83	8
	GIT parasitism	33	66	33	66	66	33	–	–	16	83	8
	Fashiolosis	40	60	60	40	80	20	–	–	20	80	6
	Coenurosis	–	100	66	33	33	66	–	–	33	66	4
	Pregnancy toxemia	–	100	–	100	–	100	–	–	100	–	1
	Wound	–	100	100	–	–	100	–	–	–	100	1
Caprine	Pasteurollo sis	66	33	33	66	55	33	11	–	44	55	31
	GIT parasitism	40	60	60	40	80	20	–	–	20	80	17
	Sheep and goat pox	66	33	33	66	33	66	–	–	66	33	10
	Contagious ecthyma	100	–	66	33	66	33	–	–	100	–	10
	Mite infestation	33	66	66	33	33	66	–	–	66	33	10
	Tick infestation	–	100	66	33	33	66	–	–	–	100	10
	Hoof over growth		100	–	100	–	100	–	–	–	100	3
	PPR	100	–	–	100	100	–	–	–	`100	–	3
	Wound	100	–	–	100	–	100	–	–	–	100	3

#### Goat diseases

In this study, goat constitutes smaller number of animals diagnosed with 29 in number. Similar to sheep, goat were also highly infected by pasteurollosis with proportion of 31%, followed by gastrointestinal parasites (31%) and sheep and goat pox and contagious ecthyma in which each disease accounted 10% proportion. The less frequently encountered problem were hoof over growth, peste des petits ruminants (PPR) and wound, with 3% proportion in all cases (Table 2).

#### Disease of equids

Back sore with proportion of 23% followed by epizootic lymphangitis (21%) were the most frequent disease encountered in which all cases were in adult groups. All the cases of epizootic lymphangitis were existed in adult horses (Table 3).

**Table 3:** Incidence of major equids diseases

Disease	Age		Sex		Species		BCS			Management system			Overall proportion (%)
	Young (%)	Adult (%)	Male (%)	Female (%)	Horse (%)	Donkey (%)	Poor (%)	Medium (%)	Good (%)	Intensive (%)	Semiintensive (%)	Extensive (%)	
Wound on the back	14	85	71	28	35	64	42	50	7	–	35	64	23
Epizootic lymphangitis	–	100	100	–	100	–	69	31	–	–	100	–	21
Lameness	12	87	75	25	62	37	50	37	12	–	62	37	13
GIT parasite	33	66	50	50	33	66	83	16	–	–	33	66	10
Tetanus	33	66	66	33	33	66	33	66	–	–	33	66	10
Anthrax	60	40	60	40	–	100	4	36	–	–	100	–	8
Respiratory disease	50	50	75	25	50	50	25	50	25	–	50	50	7
Colic	–	100	75	25	75	25	50	50	–	–	75	25	7
Hyena bite	100	–	–	100	–	100	–	50	–	–	–	100	2

Non-infectious diseases such as back sore with the proportion of 39% was the commonest equine diseases in the area. Whereas, reproductive disorders such as retained faetal membrane, dystocia are the second common health problems affecting cattle with a proportion of 16%. Next to back sore(Kusliy), colic and lameness which are locally known as Kurtset and Hinkase respectively revealed proportion of 23 and 21% respectively. Reproductive disorders and bloat were also identified as one of the health problems of cattle with proportion of 16 and 14% respectively.

This study showed that livestock diseases have a severe impact on the small holder farmer’s livelihood directly and indirectly. In previous studies, animal diseases have also been indicated as public health hazards [5]. The current clinical case study revealed that among the infectious diseases, actinomycosis (16%), mastitis (15%) and black leg (6%) were the most common diseases in the area. Due to shortage of adequate feed, cattle were subjected to deep grazing which predispose them to trauma on the oral cavity which enhances infection. Mastitis occurred with proportion of 50 and 55% in intensively managed and cross breed cattle respectively and the proportion of mastitis in semi-intensive management system and local breeds were 44 and 28% respectively. This might be due to local cattles are resistant to diseases as compared to the cross breeds and management practices is also a factor in the health status of animals. Mastitis was the major dairy cow health problem with high prevalence (16%) in Assela, Eastern Ethiopia [11]. Black leg was reported to be the most important infectious disease with prevalence of 20% in the Northern part of Ethiopia [12]. Another report showed that black leg was a common infectious disease of cattle in another region of the country [13]. Results from questionnaire survey showed that fasciolosis which is locally called ‘’Effil’’ with proportion of 19% was the common endoparasite affecting cattle in the study area. This finding agrees with other previous results in North East Ethiopia [14]. Reproductive problems such as retained fetal membrane, paraphimosis, and dystocia were among the common health problems of cattle identified. Previous results indicated that dystocia was recorded in Holeta with the prevalence rate of 7.5% [15] and 7.8% [16]. The present study also showed that pasteurollosis which is locally called “Mieta’’ was found to be the most economically important bacterial infectious disease of small ruminants with proportion of 38% in sheep and 31% in goat. The result of current study also showed that the disease highly affects young animals as compared to adults. This agrees with the previous results [17]. The second most common small ruminant infectious disease was found to be contagious ecthyma which is a viral disease of small ruminants with the proportion of 12 and 10% in sheep and goat respectively. Similar disease status was reported in different areas of the country [18]. Results from the current study revealed that tick infestation (9%), followed by mite infestation (8%) in sheep and gastro intestinal helminthiasis (17%) followed by mite infestation (10%) in goat were the most common parasitic diseases which is in consistent with previous reports [13].

The result of the questionnaire survey showed that coenurosis which is locally called ‘’Zarty’’ was among the major health problems of sheep in the study area. Similar finding was reported in Atsbi Wemberta woreda [19]. The current study showed that epizootic lymphangitis which is locally called “Defew” is the major infectious disease that affects equines with proportion of 24%. This finding agrees with previous works [20] and this disease is reported to be endemic in most African countries [21]. High incidence could be associated with sharing of infected horse harness with healthy horses which is described as major means of transmission in addition to biting flies [22]. Among the parasitic diseases affecting equines, gastro intestinal helminthiasis was the common health problem with higher proportion in donkey, 66%. Previous reports indicated that gastro intestinal helminthiasis are common health problems of equines in Wonchi area, Ethiopia [23].

### Conclusion

In conclusion, the present study has addressed the frequency of major livestock diseases in the area and this the first bases line information which can be used as evidence to start with the detail and further investigation of any livestock disease existed in the region.

## Limitations of the study

The lack of adequate laboratory facilities to diagnose all kind of animal disease was one of the limitations in this study. As a result, we are unable to guarantee the specificity and sensitivity of different test we employed in this study. There could be a high chance of mixed infections and this might affect the true map of the disease distribution in the area. In addition, only one clinic was involved in this study and only animals brought in by farmers that could afford treatment were included.

## Abbreviations

CSA: Central Statistical Authority
DVM: Doctor of Veterinary Medicine
